# Soft Palate Pleomorphic Adenoma of a Minor Salivary Gland: An Unusual Presentation

**DOI:** 10.1155/2018/3986098

**Published:** 2018-03-31

**Authors:** C. T. Forde, R. Millard, S. Ali

**Affiliations:** Department of Otolaryngology, The Royal London Hospital, Whitechapel Road, Whitechapel, London E1 1BB, UK

## Abstract

Approximately 10% of pleomorphic adenomas occur in the minor salivary glands with the palate being the most common site. Pleomorphic adenomas account for the majority of palatal tumours; however, minor salivary gland tumours have a higher risk of malignancy compared to tumours of the major salivary glands, so appropriate diagnostic evaluation should be prompt. We present a case of a 52-year-old man with a longstanding history of a soft palate pleomorphic adenoma which required excision under general anaesthetic via a mandibular swing approach. As well as the surgical approach to access this tumour; this case is unique as it is the largest soft palate pleomorphic adenoma reported in the literature. We discuss the appropriate preoperative investigations and airway considerations for this patient, as well as the factors to consider when planning operative management of palatal tumours.

## 1. Introduction

Pleomorphic adenomas (PAs) are the commonest tumour of both the major and minor salivary glands, with approximately 10% of PAs occurring in the minor salivary glands. The palate is the most common site of minor salivary gland PA, but other sites may also be affected, including the lips, cheek, floor of mouth, and nasal cavity [1].

To the best of our knowledge, this case is the largest pleomorphic adenoma of the soft palate which has been excised via a mandibular swing procedure. This case required careful preoperative planning with regards to surgical approach and airway management.

## 2. Case Presentation

A 53-year-old man presented to our department complaining of longstanding progressive dysphagia and a “hot potato” voice. He had only been managing a soft or liquid diet, and he slept with two pillows due to dyspnoea when lying flat and on exertion. He also complained of significant weight loss.

He reported that he had a lump in his mouth for thirty years and had previously presented to ENT services both in England and abroad, but he did not attend follow-up.

Clinical examination revealed a large left-sided oropharyngeal mass originating from the soft palate and extending inferiorly. The overlying mucosa was normal in appearance.

**Figure 1:**
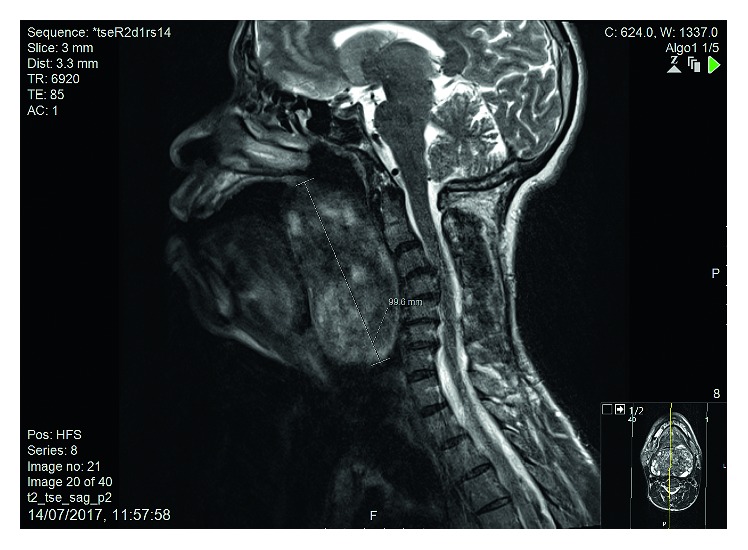
Sagittal T2-weighted MRI image demonstrating the size of the tumour in craniocaudal dimension.

Magnetic resonance imaging (MRI) demonstrated a large tumour which originated from the soft palate and extended to the larynx, measuring 9.96 cm in craniocaudal dimensions (Figure 1). Computed tomography (CT) angiography demonstrated moderate vascularity of the tumour with a hypertrophied left ascending pharyngeal artery.

**Figure 2:**
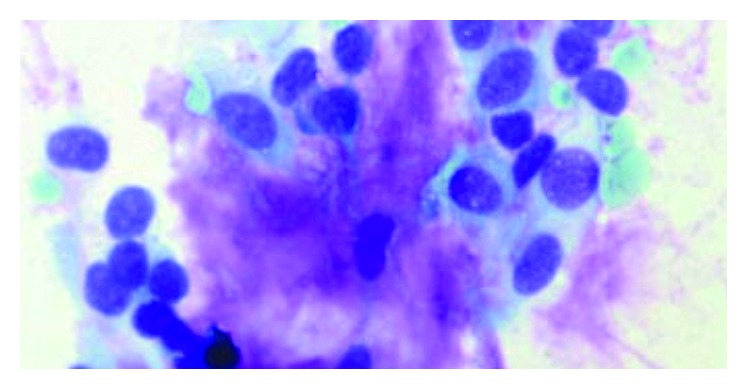
Fine-needle aspiration cytology demonstrating epithelial cells and a metachromatically staining stroma (×400 magnification).

Radiological assessment was consistent with either a myoepithelioma, a pleomorphic adenoma, or a low-grade mucoepidermoid tumour. Fine-needle aspiration cytology (FNAC) was consistent with an epithelial neoplasm, possibly of salivary gland origin (Figure 2). The multidisciplinary team decision was for surgical management.

**Figure 3:**
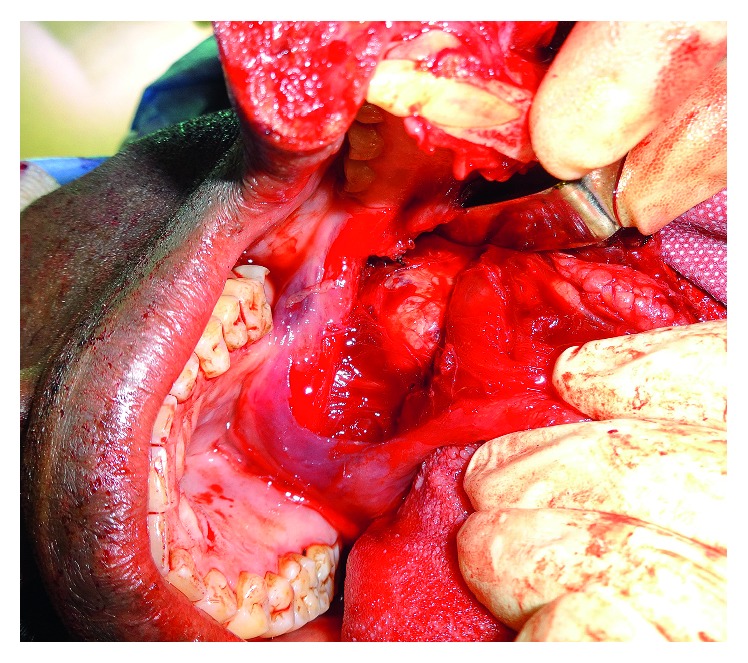
Intraoperative image demonstrating the tumour location.

**Figure 4:**
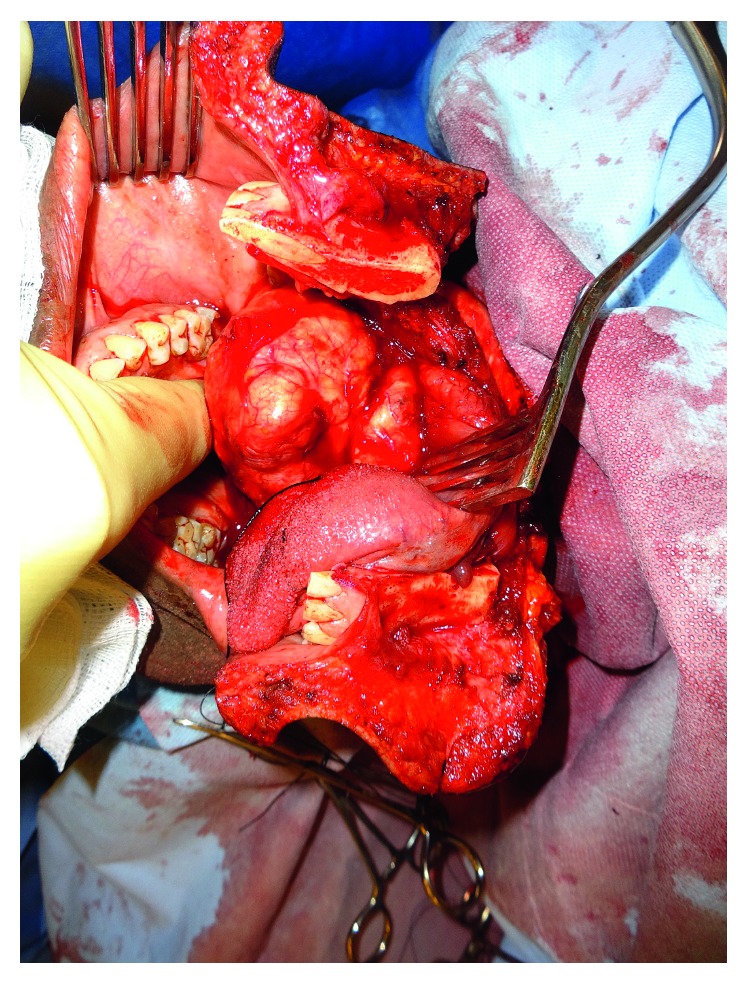
Intraoperative image demonstrating the excision of the tumour.

Due to the impossibility of oropharyngeal or nasopharyngeal intubation, he underwent a local anaesthetic tracheostomy with transnasal humidified rapid-insufflation ventilatory exchange (THRIVE). Access to the tumour was gained via a lip-split mandibulotomy and mandibular swing procedure. The whole encapsulated mass was excised from the soft palate and left parapharyngeal space via a submucosal dissection (Figures 3 and 4). The wound was then closed primarily using the abundant redundant mucosa which was sutured to the left lateral pharyngeal wall. He had an uneventful postoperative course and, following decannulation, his voice returned to normal, and he was deemed 100% intelligible scoring 0 on the GRBAS scale [2].

Video fluoroscopy demonstrated significant dysphagia due to reduction in pharyngeal bulk and failure of approximation of tongue base. Having been fed via a nasogastric tube for 6 weeks, he is currently tolerating a premashed diet with supraglottic swallowing manoeuvres. He is undergoing further swallow rehabilitation and remains under clinical surveillance.

**Figure 5:**
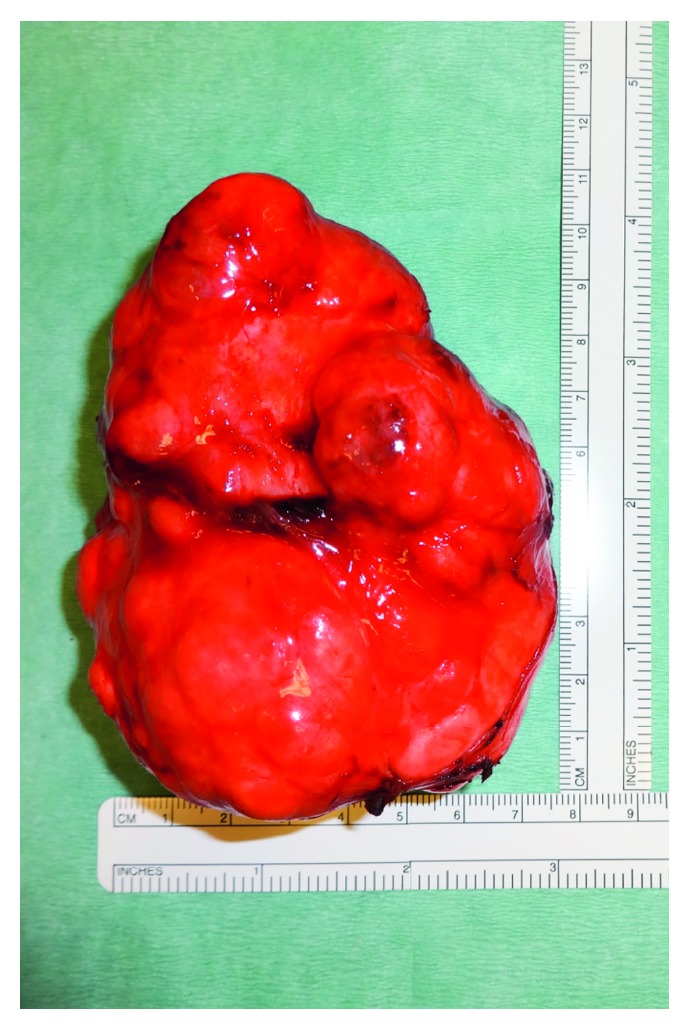
Macroscopic picture of the tumour.

**Figure 6:**
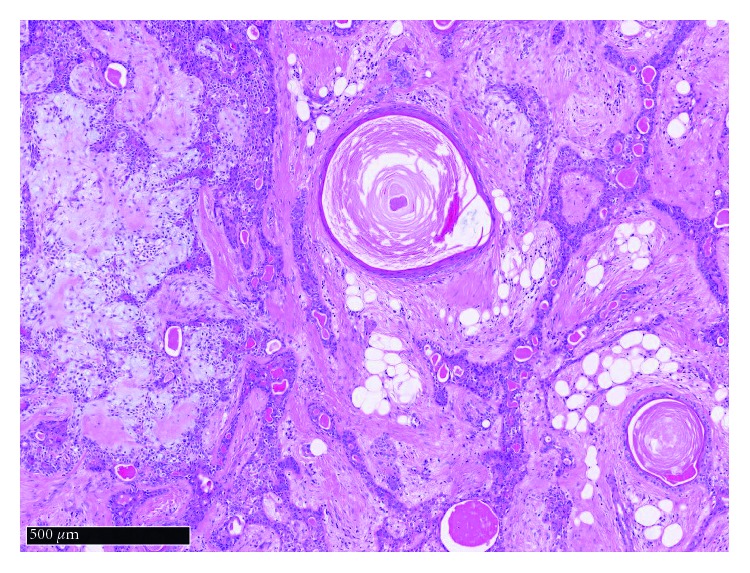
H&E stained microscopic image of the tumour (×5 magnification) demonstrating single cells in a myxochondroid stroma, keratin cysts, and tubules with adipocytic stromal change.

Histopathological examination of the excised mass demonstrated a benign encapsulated neoplasm composed of epithelium in a variety of architectural patterns consistent with a pleomorphic adenoma (Figures 5 and 6).

## 3. Discussion

Pleomorphic adenomas (PAs) are the commonest tumour of both major and minor salivary glands with about 80–90% of PA occurring in the parotid gland [3]. PA makes up approximately 40% of intraoral minor salivary gland tumours (IMSGT), and about 54% of these occur in the palate [4]. The highest incidence of palatal PA occurs between the fourth and sixth decade with a slight female preponderance [4, 5]. Previous case series have demonstrated that IMSGT are malignant in approximately 41–44% of cases [1, 4].

At 9 × 7.5 × 3.9 cm, the PA in our case represents the largest soft palate PA reported in the literature. The previous largest soft palate PA was 7 × 6 cm [6], with multiple various midsized tumours also being reported [7].

PAs demonstrate a wide variety of histological features and growth patterns. Three main histologic subgroups of pleomorphic adenoma have been identified: myxoid, cellular, and mixed (classic) type [8], almost all of which have a fibrous pseudocapsule [9].

Due to the higher risk of malignancy with IMSGTs, appropriate preoperative diagnostic investigations are appropriate. von Stempel et al. [10] suggest that MRI is useful for imaging submucosal palatal lesions like a pleomorphic adenoma as they characteristically return low T1-weighted signal and high T2-weighted signal, often with a low-signal fibrous capsule. MRI can evaluate perineural spread and provides excellent soft tissue definition. They also recommend CT scanning to assess for bony erosion, which could indicate a more aggressive lesion. In our case, a CT angiogram was also performed preoperatively due to the size of the neoplasm and the need to identify any large feeding vessels when planning the operative approach.

Intraoral ultrasound is also suggested as an adjunct [10], particularly with regard to obtaining tissue for FNAC.

Fine-needle aspiration cytology aims to differentiate benign from malignant disease, allowing more accurate counselling of the patient and aiding with surgical planning. FNAC has an accuracy of 82–97% for the preoperative diagnosis of pleomorphic adenoma [11–16]. When aspirated, PA shows combinations of three elements: ductal cells, chondromyxoid matrix, and myoepithelial cells. The diagnosis of PA is reasonably apparent when these three components are present [17]. Other studies [14, 18] have demonstrated that FNAC is reasonably accurate at excluding malignant disease in order to allow more accurate surgical planning.

Anaesthetic considerations when managing IMSGT of the palate are important, particularly given the size of the tumour we report. Due to the extent of the tumour, neither endotracheal nor fibreoptic nasal intubation was an option; therefore, the decision to proceed to tracheostomy was made. In conjunction with our anaesthetic colleagues, transnasal humidified rapid-insufflation ventilatory exchange (THRIVE) was used during the tracheostomy. THRIVE consists of high-flow humidified nasal oxygen therapy, delivered at up to 70 L/min, using OptiFlow (Fisher and Paykel Healthcare Limited, New Zealand). It has been shown to maintain oxygen saturations and extend apnoea time in patients with difficult airways [19]. THRIVE has proved to be an important adjunct during the awake tracheostomy in a previous local anaesthetic tracheostomy [20], and it proved similarly effective in this case.

The best approach for management of palatal pleomorphic adenomas is a wide excision with negative margins [21], especially as malignant transformation has been described at a rate of 1.9–23.3% [22]. Recurrence of minor salivary gland tumours is uncommon [23] but increased by intraoperative tumour spillage and inadequate surgical excision [24]. Radiotherapy is reserved for inoperable or recurrent tumours.

There are many surgical approaches possible for tumours of the soft palate, including intraoral, transcervical, and mandibulotomy and mandibular swing. The surgical approach taken depends on the size and location of the tumour, and as this tumour extended from the soft palate to the parapharyngeal space, a transmandibular approach was deemed necessary to allow adequate exposure in order to ensure complete tumour excision and control of any feeding vessels in order to prevent haemorrhage. Five considerations have been suggested by Papadogeorgakis et al. [25] when deciding the optimal surgical approach for excision of tumour of the soft palate and parapharyngeal space. They are as follows:

1. Size of the tumour
2. Suspicion of malignancy
3. Proximity and the projection of the tumour to the oropharyngeal wall or the neck
4. Vascularity
5. Relation of the tumour to the neck neurovascular bundle

Our tumour was mainly oropharyngeal, with extension to the infratemporal fossa. The inferior component was also suprahyoid. Therefore, it was felt that a lip-split mandibulotomy would give good access and delivery of the tumour. We were prepared, however, to extend the neck incision should a combined cervical approach have been necessary.

Our approach gave excellent tumour exposure. From the outset, we were able to fully mobilise and separate the tumour laterally and ensure no major feeding vessels. This approach allowed adequate excision and reduced the chance of capsule rupture and recurrence. The disadvantages of a mandibulotomy include malunion, malocclusion, injury to the mental nerve, and the cosmetic and functional consequences of the lip-split.

## 4. Conclusion

Soft palate pleomorphic adenomas of this size are rare. Appropriate diagnostic evaluation with FNAC and radiological investigations should be prompt due to the higher risk of malignancy in minor salivary glands compared to tumours of the major salivary glands.

Excisional biopsy is necessary to provide an accurate histopathological diagnosis, and careful preoperative planning should be undertaken in order assess the best surgical approach to ensure complete excision.

A comprehensive airway strategy should be planned before excision of any palatal lesion, including the use of adjuncts, such as THRIVE, if deemed appropriate.
